# Pathophysiology and new targets for therapeutic options in Chagas heart disease

**DOI:** 10.1590/0074-02760210172chgsa

**Published:** 2022-06-06

**Authors:** João Marcos B Barbosa Ferreira

**Affiliations:** 1 Universidade do Estado do Amazonas

Myocardial dysfunction represents the main cause of morbidity and mortality in patients with Chagas disease (CD). In addition, some studies suggest that heart failure caused by CD has a worse prognosis when compared to other etiologies, such as: ischemic disease and idiopathic dilated cardiomyopathy. The chapter written by Torres et al about prognosis and pending clinical challenges in Chagas heart disease (CHD) brings new perspectives on this topic.

The investigations of etiopathogenesis and pathophysiology of CHD are important to propose new therapies, in an attempt to minimise its morbidity and mortality.

The main pathophysiological mechanisms involved in CHD are microvascular dysfunction, impairment of the autonomic nervous system (ANS), direct aggression from the parasite and severe inflammatory activity. Some of these mechanisms, such as the impairment of the autonomic nervous system and the severe inflammatory activity, are also present in other cardiophathy etiologies, but are more evident in Chagas cardiopathy.^1^

Microvascular dysfunction does not seem to be an independent mechanism in the pathogenesis of chronic CHD, but it may contribute to potentiate inflammatory aggression to the myocardium. Regarding ANS involvement, there is ample evidence that CD is associated with lesions in neural structures related mainly to the parasympathetic system, but also to the sympathetic system. In the gastrointestinal tract, injuries to the parasympathetic system are essential for the development of dilations and organ dysfunction. The detection of dysautonomia in the context of CHD is important for its contribution to triggering complex arrhythmias, including sudden death. Furthermore, autonomic dysfunction has the potential to influence other metabolic and inflammatory pathways present in the pathophysiology of CHD.^1^

Another pathophysiological mechanism for CHD is direct damage by the parasite. This myocardial aggression has been observed in experimental models and in biopsy results. The presence of the parasite in the cardiac tissue of infected patients, although scarce, is relevant to the persistence of the inflammatory response during the chronic phase. Studies performed with endomyocardial biopsy demonstrate that the persistence of the parasite in patients with CHD is associated with high-grade myocarditis, and this myocarditis is associated with ventricular dysfunction. In addition to the presence of the parasite, inflammatory activity also plays a key role as a pathophysiological mechanism for CHD; it is even considered the main mechanism responsible for myocardial aggression and ventricular dysfunction in CD.^1^

These mechanisms can influence each other and also influence other pathophysiological pathways like glucose metabolism, for example. Another example of this influence is that inflammatory activity can be regulated by the autonomic function through the so-called “inflammatory reflex”. This mechanism consists of the inhibition, by the vagus nerve, of the activity of macrophages and the release of cytokines.^2^ This interrelationship between the various pathophysiological mechanisms must be studied with the aim of seeking new therapeutic proposals for this disease.

One of the pathophysiological mechanisms that needs further knowledge is the metabolic pathway. Adipose tissue exerts an important endocrine and immune action through the production of bioactive mediators called adipocytokines. There are various described adipocytokines, being produced mainly by adipocytes. Most of the adipocytokines secreted by adipose tissue correspond to adiponectin and leptin. Adipocytokines also seem to be important mediators of the inflammatory response during infections.^3^

*Trypanosoma cruzi* presents tropism for adipose tissue. In 1995, Andrade e Silva^4^ carried out studies in animal models with immunohistochemistry and electron microscopy, demonstrating that adipocytes can be infected by *T. cruzi*; it may even serve as a reservoir for possible disease reactivations. Coombs et al.^5^ carrying out a study in rats infected in the acute phase of CD, demonstrated that adipocytes infected with *T. cruzi* exhibit changes in the secretion of adipocytokines. There was a decrease in the levels of adiponectin and leptin and an increase in the levels of interleukin-6 and TNF-ɑ.^5^ Another study carried out by Nagajyothi et al.^6^ demonstrated an increase in the expression of pro-inflammatory cytokines including interleukin-6 and TNF-ɑ in cultured adipocytes infected with *T. cruzi*. Recently, Ferreira et al.^7^ demonstrated the persistence of *T. cruzi* in adipocytes of humans with chronic CHD. Changes in adipocyte function caused by Chagas infection may contribute to the pathophysiology of CHD.

There are few studies on other metabolic parameters in CD with conflicting results. Regarding glucose metabolism, there is evidence of pancreatic involvement in patients with acute and chronic CD, with an increase in the size and number of pancreatic islets, inflammatory infiltrate, fibrosis and pancreatic neuronal depopulation. Some studies demonstrate a decreased insulinemia response to glucose overload, both, oral and venous, suggesting reduced insulin secretion caused by autonomic denervation or pancreatic injury. Santos et al.^8^ reported a higher frequency of Diabetes Mellitus and hyperglycemia in women with CHD compared to controls, digestive form and indeterminate form. As an explanation, they suggested excessive sympathetic activity resulting from parasympathetic denervation and/or hypoinsulinemia resulting from functional and anatomical impairment of the pancreas.

In summary, some aspects of the pathophysiology of CHD are still unknown. CHD has specific characteristics such as cardiac denervation, exacerbated inflammatory activity and neurohormonal abnormalities that differentiate it from other heart diseases. This peculiar pathophysiology suggests that some metabolic parameters may be altered in chagasic patients. In these patients, there are few studies measuring the different adipocytokines and evaluating their role in the interrelationship between autonomic dysfunction, metabolism and inflammation. The association between metabolic parameters, inflammatory cytokines and autonomic dysfunction in patients with CD is still controversial, as it is an important area of research to be explored. This association may be important in the pathophysiology of CHD and its better understanding may open new perspectives in the study of the disease.

Besides this, the pathophysiology of CHD is well described in the chronic phase of CD, but the study of cardiac involvement in the acute phase of this disease can also elucidate pathophysiological mechanisms that influence the progression to CHD. The cases of acute Chagas disease (ACD) have become more frequent due to outbreaks of oral transmission mainly in the Amazon region. Specifically in the acute phase of CD, there are publications demonstrating cardiac involvement mainly through electrocardiogram and echocardiogram. Some of these studies evaluated the medium or long-term follow-up after treatment of the acute phase.^9^ ^,^ ^10^ However, further studies are needed in on the various mechanisms of ACD such as involvement of the ANS, profile of inflammatory cytokines, imaging studies with magnetic resonance in the assessment of myocardial injury and evaluation of metabolic mediators such as adipocytokines and insulin.

Better knowledge of these new pathophysiological pathways, even in the acute phase, may be useful in improving the evolution of this still-neglected disease, with high prevalence and mortality.

Comments on the article: Torres RM, Correia D, Nunes MCP, Dutra WO, Talvani A, Sousa AS, et al. Prognosis of chronic Chagas heart disease and other pending clinical challenges. Mem Inst Oswaldo Cruz. 2022; 117: e210172.
